# Technology is the New Normal for Individuals Aging-In-Place With Disabilities

**DOI:** 10.1093/geroni/igab046.1640

**Published:** 2021-12-17

**Authors:** Travis Kadylak, Susy Stark

## Abstract

Technology designers often exclude individuals aging with diverse needs, capabilities, and disabilities from engaging in the design process, which can hinder the usability and usefulness of emerging technologies. In this symposium, investigators report on research and development efforts aimed at understanding the needs of, and developing supportive technologies for, people aging with long-term disabilities. This symposium features projects from the Rehabilitation Engineering Research Center on Technologies to Support Aging-in-Place for People with Long-Term Disabilities (RERC TechSAge), which is an interdisciplinary collaboration between Georgia Tech and the University of Illinois at Urbana-Champaign. First, Bayles et al. will discuss findings from the Aging Concerns, Challenges, and Everyday Solution Strategies II study, focused on Deaf older adults’ use of technologies as solution strategies for common everyday challenges. Next, Mitzner et al. will highlight the development of an evidence-based group exercise intervention (Tellewellness Tai Chi for Arthritis) aimed at promoting both physical exercise and social interaction for older adults with long-term mobility disabilities. Kadylak et al. will describe how voice-activated digital assistants can support older adults aging with mobility disabilities by reporting on findings from a longitudinal demonstration project with older adults in assisted and independent living communities. Exploring the potential for smartbathroom technology to promote aging in place, Sanford et al. will discuss how smartbathroom sensor data can be analyzed and vizualized to identify ways to communicate insight from sensor data to improve training of occupational therapy practitioners. Susy Stark from Washington University will serve as the discussant for the symposium.

